# Recurrent venous thromboembolism and clot distribution in COVID-19 infection: A review by variant type

**DOI:** 10.1371/journal.pone.0331283

**Published:** 2025-09-22

**Authors:** Laura Saldivar, Tannaz Rajabi, Wei Li, Samia Lopa, Dominick Roto, Jeffrey Bruckel, Daniel Lachant, Mark Marinescu

**Affiliations:** 1 Department of Internal Medicine-Pediatrics, University of Rochester Medical Center, Rochester, New York, United States of America; 2 University of Rochester School of Medicine and Dentistry, Rochester, New York, United States of America; 3 Department of Biostatistics and Computational Biology, University of Rochester Medical Center, Rochester, New York, United States of America; 4 Department of Pulmonary & Critical Care Medicine, University of Rochester Medical Center, Rochester, New York, United States of America; 5 Department of Cardiology, University of Rochester Medical Center, Rochester, New York, United States of America; Ataturk University Faculty of Medicine, TÜRKIYE

## Abstract

**Research question:**

Do PE distribution and rates of recurrent VTE differ between COVID-19 (CAPE) and non-COVID-19 (NCAPE) patients and among COVID-19 variants?

**Study design and methods:**

A single-center retrospective chart review of 547 patients with PE admitted from January 2020-October 2022 was conducted. 470 patients did not have COVID-19 infection on admission. 77 patients had COVID-19 infection with 17, 27, and 33 admissions occurring during Alpha, Delta, and Omicron predominance, respectively. Imaging reports, follow-up, and recurrent VTE incidence were extracted. Central clot was defined as saddle or mainstem PE. Clot distribution was classified as central, peripheral, or both. Recurrent VTE was examined at 3 and 6 months.

**Results:**

NCAPE and CAPE patients had similar patterns of clot distribution overall (P = 0.34, 0.48, & 0.82 for central, peripheral, and both, respectively). Of CAPE patients with solely peripheral PE distribution (N = 49), Omicron comprised 78.8% (N = 26, P = 0.01). Recurrent VTE occurred in 45 patients. The cumulative proportion of recurrent VTE or death did not differ significantly by COVID-19 status (P = 0.12).

**Conclusions:**

- COVID-19 infection is associated with VTE events which appear to vary in frequency and location among the different variants (Alpha, Omicron, and Delta).
- VTE risk is highest in the 3 months following COVID-19 infection.
- Recurrent rate of VTE is similar between patients with COVID-19-associated and non-COVID-19-associated PE.
- The VTE risk and clot distribution of COVID-19 is evolving over the years as the virus becomes endemic in the United States and warrants further study.

## Introduction

SARS-CoV-2 (COVID-19) infection is a known risk factor for venous thromboembolism (VTE) [1,2]; however, the clinical characteristics of pulmonary embolism (PE) in COVID-19, such as location (central vs. peripheral) or recurrence rates, remain unclear [3–12]. Similarly, data on PE incidence among the specific variants of COVID-19 is also limited, with some studies suggestive of lower incidence of PE in Omicron infection compared to earlier variants [13]. Total clot distribution in patients with COVID-19 represents an important area of further study since thrombus distribution and burden have been linked to increased mortality in this population [14]. Recurrent VTE risk is of particular concern as a large study recently noted that these patients can have up to a 5-fold higher rate of major bleeding than VTE recurrence during the first 90 days of anticoagulation [15]. This risk of bleeding must be weighed against studies showing risk of VTE recurrence. A new prospective, multicenter trial by the RIETE Investigators showed that in patients with COVID-19-associated VTE treated with 3 months of anticoagulation, the recurrent VTE and case-fatality rates were low, suggesting that long-term anticoagulation may not be needed in this population [16].

This study aims to compare PE location, PE distribution, and rates of recurrent VTE between patients with COVID-19-associated pulmonary embolism (CAPE) and non-COVID-19-associated pulmonary embolism (NCAPE), and to stratify these results by variant type. We hypothesized that CAPE patients would have more central or combined (both central and peripheral) PE distribution given COVID-19’s pro-inflammatory response causing endothelial and hemostatic activation [17]. We also hypothesized that CAPE patients would have lower rates of recurrent VTE given the provoked nature of their clots, compared to NCAPE patients who may have experienced unprovoked clots.

### Study design and methods

This was a single-center retrospective observational study with IRB exemption granted by the University of Rochester Office for Human Subject Protection. Informed consent requirements were waived by the Office for Human Subject Protection given the retrospective nature of the study. A total of 1,285 charts were identified using billing codes for PE diagnoses (I26, I26.0, I26.09, and I26.9) associated with admissions to our institution between January 2020 and October 2022. On further manual chart review (conducted between January 2023 and March 2023) of the charts identified by billing codes, a total of 547 patients with confirmed PE (as noted on chest computed tomography angiography) were identified. Individual, identifiable patient information was initially available to the data entry team (Laura Saldivar, Tannaz Rajabi, and Wei Li) to facilitate manual chart review to confirm the accuracy of the electronic medical record data pull results. All data was then fully de-identified prior to statistical analysis (conducted by Samia Lopa). Patients were considered to be COVID-positive if they had a positive test on admission or a positive test (home or PCR) documented in the 30 days preceding admission. Centers for Disease Control (CDC) data was used to identify the variant types in circulation in our institution’s city by date as specific variant data was not available. CT angiography imaging data (using radiology reports), date of last medical follow-up/death from any cause, and incidence of recurrent VTE were extracted manually. Use of anticoagulation was defined as being prescribed and actively taking an anticoagulant (such as a direct oral anticoagulant or warfarin) in the days preceding admission per patient report within history of present illness documentation. Central clot was defined as saddle or mainstem PE; lobar, segmental and subsegmental PEs were considered peripheral. Clot distribution was classified by the presence of central, peripheral, or combined (central and peripheral) PE. Recurrent VTE, examined at 3 and 6 months, was defined as PE that was new or worsening based on an increased clot size or distribution compared to prior scans. Kaplan-Meier survival curves and log-rank tests were used to estimate the cumulative proportion of recurrent VTE and the combined incidence of recurrent VTE or death from any cause. Statistical analysis was performed using statistical packages SAS and R.

## Results

The study population (Table 1) included a total of 547 patients with confirmed PE. These patients were classified as having either NCAPE (n = 470) or CAPE (n = 77). Of the 77 COVID-positive patients, 17 PEs occurred during Alpha variant predominance, 27 during Delta variant predominance, and 33 during Omicron variant predominance in our region. Overall, the two populations were similar in age, body mass index, prior history of VTE, and use of anticoagulation at time of admission. There were slightly more females in the NCAPE group (52.6%) compared to the CAPE group (39%). Prior history of cancer was more common in the NCAPE group (30.2% vs. 16.9%), though rates of active cancer (defined as currently undergoing chemotherapy and/or radiation at the time of index admission) were similar (19.8% vs. 10.4%). History of tobacco use and active smoking were more common among the NCAPE population (52.3% vs. 39% and 15.7% vs. 3.9%, respectively).

**Table 1:** Patient characteristics by COVID-19 status.

Variable	All N (%) 547 (100%)	NCAPE N(%) 470 (86%)	CAPE N(%) 77 (14%)	P-value
Age (Years)	65.0 (54.0: 75.0)	65.0 (54.0: 75.0)	61.0 (54.0: 70.0)	0.08
Gender (Female)	277 (50.6%)	247 (52.6%)	30 (39.0%)	0.033
BMI (kg/m^2^)	29.8 (25.3: 35.9)	29.7 (25.4: 35.9)	30.0 (25.2: 36.1)	0.98
Prior history of DVT/PE	81 (14.8%)	72 (15.3%)	9 (11.7%)	0.48
Prior History of Cancer	392 (71.7%)	328 (69.8%)	64 (83.1%)	0.019
Active Cancer	101 (18.5%)	93 (19.8%)	8 (10.4%)	0.06
Ever Smoker	276 (50.5%)	246 (52.3%)	30 (39.0%)	0.032
Active Smoker	77 (14.1%)	74 (15.7%)	3 (3.9%)	0.007
On AC on admission	49 (9.0%)	43 (9.2%)	6 (7.8%)	0.84

For continuous variables, medians reported with confidence intervals.

Overall, NCAPE and CAPE patients had similar patterns of clot distribution (central, peripheral, or both) (Table 2). Of CAPE patients with solely peripheral PE distribution (N = 49) stratified by variant type, Omicron comprised 78.8% (N = 26), compared to Alpha (N = 6) and Delta (N = 17) (P = 0.01). Omicron had a higher incidence of peripheral clot distribution compared to Alpha and Delta variants. The largest branch affected across both NCAPE and CAPE patients was overall similar (Table 3).

**Table 2:** Clot Distribution By COVID-19 and Variant Status.

COVID-19 Status	Variant	Central [N (%)]	Peripheral [N (%)]	Both [N (%)]
NCAPE [N = 470]	N/A	20 (4.26)	279 (59.36)	171 (36.38)
CAPE [N =77]	All [N = 77]	1 (1.3)	49 (63.64)	27 (35.06)
	Alpha [N = 17]	1 (5.88)	6 (35.29)*	10 (58.82)**
	Delta [N = 27]	0 (0)	17 (62.96)*	10 (37.04)**
	Omicron [N = 33]	0 (0)	26 (78.79)*	7 (21.21)**

P-values are from Fisher’s Exact/Chi-square test as appropriate. *P = 0.01 among variants in patients with peripheral PE distribution. **P = 0.03 among variants in patients with both peripheral and central PE distribution.

**Table 3:** Largest PE Site by COVID-19 and Variant status.

COVID-19 Status	Variant	Saddle [N (%)]	Mainstem [N (%)]	Lobar [N (%)]	Segmental [N (%)]	Subsegmental [N (%)]
NCAPE [N = 470]	N/A	63 (13.4)	128 (27.23)	108 (22.98)	142 (30.21)	29 (6.17)
CAPE [N = 77]	All [N = 77]	10 (12.99)	18 (23.38)	20 (25.97)	24 (31.17)	5 (6.49)
	Alpha [N = 17]	4 (23.53)*	7 (41.18)	2 (11.76)	4 (23.53)	0 (0)
	Delta [N = 27]	5 (18.52)*	5 (18.52)	6 (22.22)	8 (29.63)	3 (11.11)
	Omicron [N = 33]	1 (3.03)*	6 (18.18)	12 (36.36)	12 (36.36)	2 (6.06)

P-values are from Fisher’s Exact/Chi-square test as appropriate. *P = 0.04 for saddle emboli among variants.

**Fig 1:**
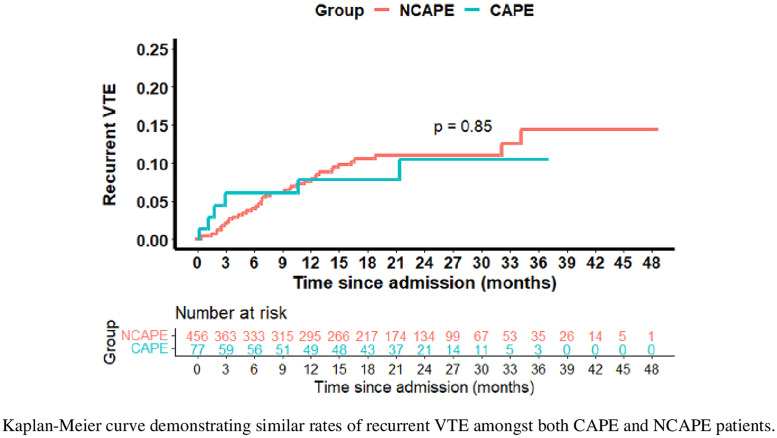
Cumulative proportion of recurrent VTE by COVID-19 status. Kaplan-Meier curve demonstrating similar rates of recurrent VTE amongst both CAPE and NCAPE patients.

Recurrent VTE analysis was limited to 533 patients (Fig 1). In this population, 45 patients had recurrent VTE (6 CAPE and 39 NCAPE). Recurrent VTE (Tables 4 and 5) or death from any cause (Table 6) did not differ significantly by COVID-19 status (P = 0.12), though Delta had a significantly higher rate of recurrent VTE (16.5%) and death (28.9%) compared to the other variants. All of the recurrent VTEs in CAPE patients in our 3–6 month study window occurred within the first 3 months of admission (Table 4, N = 4). When follow-up was limited to 6 months, the incidence of recurrent VTE over time was somewhat different between CAPE and NCAPE patients, however the difference was not statistically significant (P = 0.39, S1a Fig). Patients with a prior history of VTE had similar rates of recurrent VTE as those without this history (Table 4, P = 0.87).

**Table 4:** Estimated cumulative percentage of recurrent VTE at specific time points.

Group	3 Months	6 Months
Overall	2.6% (1.5%, 4.6%)	4.3% (2.8%, 6.7%)
NCAPE	2.1% (1%, 4.1%)	4% (2.4%, 6.6%)
CAPE	6% (2.3%, 15.3%)	6% (2.3%, 15.3%)
Alpha	5.9% (0.9%, 35%)	5.9% (0.9%, 35%)
Delta	16.5% (5.6%, 43.3%)	16.5% (5.6%, 43.3%)
Omicron	0% (0%, 0%)	0% (0%, 0%)
Prior History of DVT/PE		
No	2.6% (1.4%, 4.7%)	4.2% (2.6%, 6.8%)
Yes	3.2% (0.8%, 12.1%)	4.9% (1.6%, 14.4%)

Percent of patients with VTE events reported with confidence intervals listed in parentheses.

**Table 5:** Estimated cumulative percentage of recurrent VTE among COVID-19 patients at specific time points.

Prior History of DVT/PE	3 Months	6 Months
No	7% (2.7%, 17.5%)	7% (2.7%, 17.5%)
Yes	0% (0%, 0%)	0% (0%, 0%)

Percent of patients with VTE events reported with confidence intervals listed in parentheses.

**Table 6:** Estimated cumulative percentage of death at specific time points.

Group	3 Months	6 Months
Overall	14.4% (11.7%, 17.7%)	18.2% (15.1%, 21.8%)
NCAPE	14.8% (11.8%, 18.4%)	19.1% (15.8%, 23.1%)
CAPE	12.3% (6.6%, 22.4%)	12.3% (6.6%, 22.4%)
Alpha	0% (0%, 0%)	0% (0%, 0%)
Delta	28.9% (14.9%, 51.3%)	28.9% (14.9%, 51.3%)
Omicron	6.3% (1.6%, 22.7%)	6.3% (1.6%, 22.7%)
Prior History of DVT/PE		
No	13.8% (11%, 17.4%)	18% (14.7%, 21.9%)
Yes	17.8% (10.9%, 28.2%)	19.1% (12%, 29.7%)

Percent of deceased patients with confidence intervals listed in parentheses.

## Discussion

We found similar rates of recurrent VTE between CAPE and NCAPE populations, with all CAPE VTE events occurring within the 3 months following index hospitalization. We also found similar clot distribution in both populations. Of those with COVID-19, peripheral-only clot distribution was seen more commonly in the Omicron variant, whereas combined clot distribution was seen more commonly in those affected by the Delta or Alpha variants.

The mechanism of hypercoagulability in COVID-19 infection is postulated to be secondary to the interaction of the spike glycoprotein with ACE2 receptors present in the host cell surface mediating the entry of the SARS-CoV-2 virus. This results in internal injury inside the vascular wall with direct or indirect activation of the coagulation and clotting cascades [18]. Provoked clots include those caused by transient risk factors (surgery, trauma, immobility, pregnancy or puerperium, hormonal medication use, or COVID-19 infection), while unprovoked clots may be secondary to an underlying hypercoagulable disorder such as protein C/S deficiency, antiphospholipid antibody syndrome, prothrombin gene mutation, or other undefined hypercoagulable states.

The CAPE and NCAPE clot locations (central vs. peripheral) in the study population were overall similar with a trend toward clot distribution becoming more peripheral in nature as the virus evolved. This is potentially due to the mutations in the pathogenicity and virulence of the various COVID-19 variants, which may affect the endothelial response and coagulation pathways differently [19]. Vaccinations or prior exposures may have played a crucial role in blunting the severity of COVID-19 by enhancing the immune system’s ability to quickly recognize and combat the virus, thereby reducing the incidence of severe cases and associated complications like thrombosis [20]. Lastly, later on in the course of the COVID-19 pandemic, those who were going to manifest with the highest PE distribution may have already experienced their index events. Research on COVID-19-associated clot distribution and burden is limited at the time of submission, with one study noting a correlation between elevated Mastora scores and increased mortality in COVID-19 patients [14].

Recurrent VTE analysis was limited to 533 patients as 14 patients did not have dates of further medical follow-up data available. Our results suggest that the highest incidence of recurrent VTE is in the 3 months following index hospitalization for COVID-19. This finding is supported by the evolving literature [15,16]. This is presumed to be related to the pro-inflammatory state of the infection; once this pro-inflammatory state resolves, the rate of recurrent VTE in CAPE patients appears to return to that of the non-COVID-19 population.

There were a large number of charts initially identified by billing codes that, when manually reviewed, did not have evidence of an acute PE during the admission window (57% of charts with PE as a billing code did not have an acute PE during the index hospitalization). We believe the reason for this may be multifactorial and related to PE-related ICD-10 codes being inappropriately pulled from prior hospitalizations, likely due to association with imaging orders such as computed tomography angiography, refills of prior anticoagulant medications at the time of admission/discharge, or error. These findings highlight the limitations of utilization of claims data and demonstrate the need to understand the accuracy of claims data for defining a cohort, particularly as more studies begin to utilize large electronic databases and billing code analysis.

This study has several limitations. First, specific PCR testing for variants was not available; as such, CDC data was used for the county of our institution. Although we observed interesting differences across CAPE while different variants were predominantly circulating, these findings must be interpreted cautiously as our sample size for the CAPE group is not large enough to draw a definitive conclusion about the differences across variants. Any differences observed between variants might also represent the effects of public health measures, vaccines, and therapies during the course of the pandemic rather than a true physiologic difference. Provoking risk factors (such as immobility, recent surgery, and history of hormonal treatment) were found to be inconsistently documented by providers and thus were unable to be included in the analysis. However, these results could still provide background for future studies in these patient populations. It should also be noted that particularly early on in the pandemic some patients affected by the Alpha variant may have not undergone CT scans to decrease hospital-wide exposure to COVID-19. These patients may have instead been treated based on clinical suspicion alone. Second, vaccination status was not obtained during this study and may have led to reduced rates of COVID-19 infection as well as recurrent VTE events. Third, this study is a single-institution retrospective analysis and recurrent VTE analysis was only able to be performed using information available in the medical record. Fourth, patient adherence to prescribed anticoagulation regimens was not specifically monitored in this study and patients may have experienced lapses in pharmacotherapy due to insurance/cost-related or personal factors that influenced their risk for recurrent VTE. However, we would not anticipate that these barriers would vary significantly between the two patient populations (CAPE and NCAPE). Some cases of CAPE may have been classified as NCAPE due to patients not performing COVID-19 testing at home with negative testing upon hospital admission (despite clinical infection in the preceding 30 days). Lastly, imaging reports were analyzed for patient characterization and the imaging did not undergo blinded radiologist review for confirmation of accuracy.

## Conclusions

CAPE and NCAPE patients had a similar clot distribution with a trend toward less central PE occurring with the evolution of the COVID-19 virus. Despite COVID-19’s role as a provoking cause of VTE, rates of recurrent VTE were similar between CAPE and NCAPE patients. VTE events associated with COVID-19 infection all appeared in the 3 months following hospital admission, suggesting that VTE risk is highest during this time frame. These findings support the continued use of standard anticoagulation protocols in COVID-19 patients and demonstrate that the provoking nature of the infection does not necessarily imply a higher recurrence rate of thrombotic events. Guidelines on the duration and intensity of anticoagulation in recovered COVID-19 patients should be reviewed to reduce the risk of potential over-treatment.

Ultimately, this study reveals the need for ongoing research into the evolving nature of COVID-19 and other respiratory illnesses as they relate to the long-term effects on thrombosis. The VTE risk and clot distribution of COVID-19 is evolving over the years as the virus becomes endemic in the United States and warrants further study.

## Supporting information

S1 Fig Recurrent VTE by COVID-19 status 6 months post-hospitalization. Kaplan-Meier curve demonstrating recurrent VTE rate by COVID-19 status through 6 months post-hospitalization.
(TIF)
