# Supplementary figures and images for: Recurrent venous thromboembolism and clot distribution in COVID-19 infection: A review by variant type
Source: PLoS One. 2025 Sep 22;20(9):e0331283. doi: 10.1371/journal.pone.0331283 (PMC12453200; doi:10.1371/journal.pone.0331283)

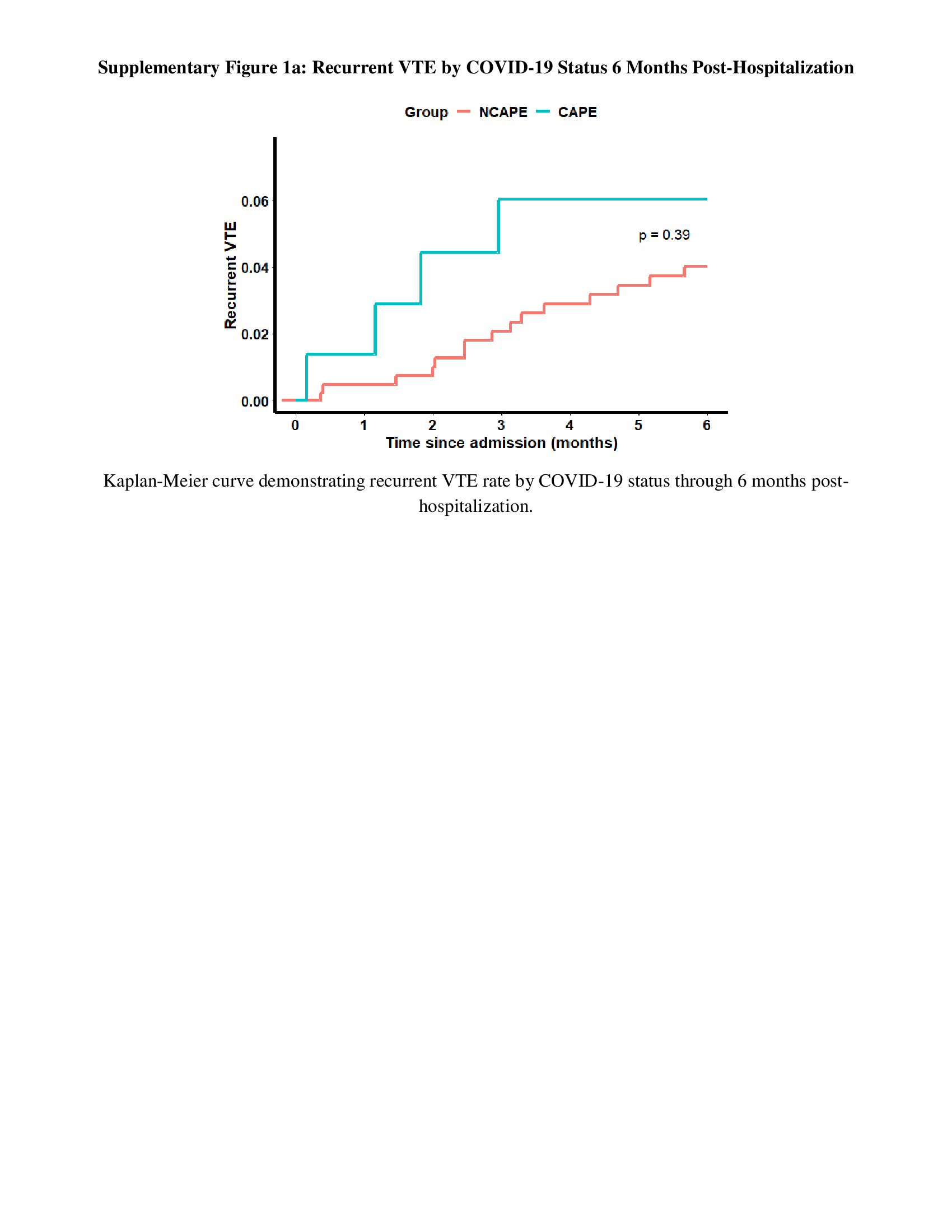

Supplement: S1 Fig — Kaplan-Meier curve demonstrating recurrent VTE rate by COVID-19 status through 6 months post-hospitalization. (TIF) [file pone.0331283.s001.tif]
